# Ageism and Its Impact on Older Persons’ Health

**DOI:** 10.1093/geroni/igaa057.2330

**Published:** 2020-12-16

**Authors:** Becca Levy

**Affiliations:** Yale School of Public Health, Woodbridge, Connecticut, United States

## Abstract

Ageism has been called a silent epidemic. The extent to which ageism impacts the health of older persons in different countries was not well understood. In this presentation we will focus on the reach of ageism including negative age beliefs, on older individuals’ health. In an exhaustive systematic review, we found that ageism influenced older individuals in 45 countries and 11 health domains, with the prevalence of significant findings increasing over time (p < .001). In this presentation, we will also explore the mechanism by which this impact occurs and steps that can be taken to address this epidemic.

